# Techno-productive, environmental, and epidemiological trajectories in the Amazon, Brazil: a path towards synthesis

**DOI:** 10.1590/0102-311XEN227323

**Published:** 2025-03-24

**Authors:** Cláudia Torres Codeço, Antonio Miguel Vieira Monteiro

**Affiliations:** 1 Programa de Computação Científica, Fundação Oswaldo Cruz, Rio de Janeiro, Brasil.; 2 Instituto Nacional de Pesquisas Espaciais, São José dos Campos, Brasil.

Amazonian socioenvironmental abundance hides a great fragility, exposed to the world by the extreme drought of 2023 when rivers dried up, the poor sandy soil was exposed, animals died in the warm water, hunger set in, and the sky became darkened by wildfire smoke. The rapid environmental and social transformation of this biome is intrinsically related to land demand for producing agricultural commodities, which together with illegal logging and mining have caused bloody conflicts, as well as biodiversity losses, environmental contamination and the risk of new diseases emerging ^1^ ^,^ ^2^. Traditional production systems, including agroextractivism and small-scale livestock farming, survive in the region under great risk. These sectors, although lacking economic incentives compared to agribusiness, remain crucial for a substantial part of the population and also for preserving the forest and its ecosystem services. Development models for the Amazon have been debated, but the metrics and indicators available tend to favor hegemonic strategies and make current and local solutions that have evolved within Amazon unfeasible ^3^. One consequence has been the inadequacy of public policies both to meet the needs of traditional populations and to meet novel needs that arise in the transformed space.

This Supplement describes the theoretical, methodological and analytical outcomes of the Trajectories Project, developed from the perspective of Knowledge Synthesis Science. A Synthesis Center aims to promote an interdisciplinary environment that fosters the integration of theories, methods and data at different spatial or temporal scales to counteract scientific specialization. When successful, synthesis brings together topics, specialties, or disciplines in innovative ways, opening up new spheres of research and innovating in tackling socioenvironmental challenges ^4^. Within the creative and favorable environment provided by the first Brazilian Center for Synthesis on Biodiversity and Ecosystem Services (SinBiosis, of the Brazilian National Research Council − CNPq, acronym in Portuguese), researchers from various Brazilian research institutions came together to reflect on the following guiding questions: How are different socio-environmental trajectories related to human and environmental health in the Amazon? In which scenarios are human health and environmental health simultaneously amplified by the same public policies? In which scenarios are public policies needed to reduce the costs of land use change to the environment and/or local communities?

The initial theoretical discussions, summarized in Codeço et al. ^5^, seek to understand the environment and health based on the concept of techno-productive trajectory, which is the classification of the Amazonian rural economy proposed by economist Professor Francisco Costa ^6^. Different techno-productive trajectory define specific relations between production and nature that emerge from logics, knowledge and technologies of production that do or do not incorporate an ecological context into their processes. These relations with nature, in turn, are associated with distinct epidemiological landscapes. In this Supplement, the reader will find a statement by Professor Costa on the roots of the theory of rural techno-productive trajectories in the Amazon. This theory served as the basis for creating a database for the municipalities in the Brazil’s Legal Amazon, which relates techno-productive trajectory to environmental indicators of landscape use and transformation, epidemiological indicators that measure climate-sensitive diseases, and socioeconomic indicators adapted to the rural and urban reality of the Amazon ^7^. The articles in this Supplement deepen the analysis of these indicators on different fronts and from different perspectives, bringing together the knowledge of researchers with experience in health, the environment, economy and society, to generate a more integrated and consistent narrative to explain/change the scenarios affecting ecosystems and health in the Amazon.
