# Supplementary material for: Techno-productive, environmental, and epidemiological trajectories in the Amazon, Brazil: a path towards synthesis
Source: Cad Saude Publica. 2025 Mar 24;41(Suppl 1):e00227323. doi: 10.1590/0102-311XEN227323 (PMC11940865; doi:10.1590/0102-311XEN227323)
Supplement: Translation

# Translation: Trajetórias tecnoprodutivas, ambientais e epidemiológicas na Amazônia, Brasil: caminho de síntese

**DOI:** 10.1590/0102-311XPT227323

**Authors:** Cláudia Torres Codeço, Antonio Miguel Vieira Monteiro

**Affiliations:** 1 Programa de Computação Científica, Fundação Oswaldo Cruz, Rio de Janeiro, Brasil.; 2 Instituto Nacional de Pesquisas Espaciais, São José dos Campos, Brasil.

A riqueza socioambiental da Amazônia, Brasil, esconde uma grande fragilidade, que foi exposta ao mundo na seca extrema de 2023 quando os rios se esgotaram, o solo arenoso e pobre ficou exposto, os animais morreram na água morna, a fome se instalou e o céu se tornou fumaça. A acelerada transformação ambiental e social desse bioma está intrinsecamente relacionada à demanda por terras para produção de *commodities* agrícolas que, junto à exploração ilegal de madeira e as atividades de mineração, têm causado conflitos sangrentos, perdas de biodiversidade, contaminação ambiental e risco de emergência de novas doenças ^1^ ^,^ ^2^. Os sistemas tradicionais de produção, incluindo atividades agroextrativistas e pecuária de pequena escala, persistem na região, mas estão em risco. Esses setores, embora carentes de incentivos econômicos em comparação ao agronegócio, continuam sendo cruciais para uma parte substancial da população e também para a preservação da floresta e seus serviços ecossistêmicos. Modelos de desenvolvimento para a Amazônia vêm sendo debatidos, porém as métricas e indicadores disponíveis tendem a favorecer as estratégias hegemônicas e invizibilizar soluções que já existem e evoluíram na região ^3^. Uma consequência tem sido a inadequação das políticas públicas para atender as necessidades das populações tradicionais e as novas necessidades que surgem no espaço transformado.

Este Suplemento descreve os resultados teóricos, metodológicos e analíticos do Projeto Trajetórias, desenvolvido numa perspectiva de Ciência de Síntese de Conhecimento. A proposta de um Centro de Síntese é promover um ambiente interdisciplinar que favoreça a integração de teorias, métodos e dados em diferentes escalas espaciais ou temporais, visando contrapor a especialização científica. Quando bem sucedida, a síntese reúne tópicos, especialidades ou disciplinas de maneiras inovadoras, abrindo novas esferas de investigação e inovando no enfrentamento de desafios socioambientais ^4^.

Dentro do ambiente criativo e favorável proporcionado pelo primeiro Centro de Síntese em Biodiversidade e Serviços Ecossistêmicos (SinBiose, iniciativa do Conselho Nacional de Desenvolvimento Científico e Tecnológico − CNPq), reuniram-se pesquisadores de várias instituições de pesquisa brasileiras para se pensar as seguintes perguntas norteadoras: Como diferentes trajetórias socioambientais estão relacionadas à saúde humana e ambiental no bioma Amazônico? Em que cenários a saúde humana e a saúde ambiental são amplificadas simultaneamente pelas mesmas políticas públicas? Em que cenários políticas públicas são necessárias para reduzir os custos para o meio ambiente e/ou para as comunidades locais decorrentes de mudanças de uso da terra?

As discussões teóricas iniciais, sintetizadas em Codeço et al. ^5^, buscam ver o ambiente e a saúde a partir do conceito de trajetória tecnoprodutiva, que é a classificação da economia rural Amazônica proposta pelo professor e economista Francisco Costa ^6^. Diferentes trajetórias tecnoprodutivas definem relações específicas entre produção e natureza, emergentes de lógicas, conhecimentos e tecnologias de produção que incorporam ou não um contexto ecológico em seus processos. Por sua vez, essas relações com a natureza estão associadas a paisagens epidemiológicas distintas. Neste Suplemento, o leitor encontra um depoimento do professor Costa sobre as origens da teoria das trajetórias tecnoprodutivas rurais da Amazônia, que serviu de fundamento para a produção de uma base de dados para os municípios da Amazônia Legal que relaciona as trajetórias tecnoprodutivas com indicadores ambientais de uso e transformação da paisagem, indicadores epidemiológicos que medem doenças sensíveis ao clima e indicadores sócioeconômicos adaptados para a realidade rural e urbana da Amazônia ^7^. Os artigos deste Suplemento aprofundam as análises desses indicadores em diferentes frentes e olhares, reunindo o conhecimento de pesquisadores com experiência em saúde, ambiente, economia e sociedade para gerar uma narrativa mais integrada e consistente, visando explicar/modificar os cenários que afetam os ecossistemas e a saúde na Amazônia.

# Translation: Trayectorias tecnoproductivas, ambientales y epidemiológicas en la Amazonia, Brasil: camino de síntesis

**DOI:** 10.1590/0102-311XES227323

**Authors:** Cláudia Torres Codeço, Antonio Miguel Vieira Monteiro

**Affiliations:** 1 Programa de Computação Científica, Fundação Oswaldo Cruz, Rio de Janeiro, Brasil.; 2 Instituto Nacional de Pesquisas Espaciais, São José dos Campos, Brasil.

La riqueza socioambiental de la Amazonia, Brasil esconde una gran fragilidad, expuesta al mundo en la extrema sequía del 2023, cuando los ríos se secaron, el suelo arenoso y pobre quedó expuesto, los animales murieron en el agua templada, el hambre se apoderó de ellos y el cielo se convirtió en humo. La acelerada transformación ambiental y social de este bioma está intrínsecamente relacionada con la demanda de tierras para la producción de commodities agrícolas, que junto con la explotación ilegal de madera y las actividades de minería han provocado conflictos sangrientos, además de pérdida de biodiversidad, contaminación ambiental y riesgo de aparición de nuevas enfermedades ^1^ ^,^ ^2^. Los sistemas de producción tradicionales, incluidas las actividades agroextractivas y la ganadería en pequeña escala, persisten en la región, pero están en riesgo. Estos sectores, aunque carecen de incentivos económicos en comparación con el agronegocio, siguen siendo cruciales para una parte sustancial de la población y también para la preservación del bosque y de sus servicios ecosistémicos. Se vienen discutiendo modelos de desarrollo para la Amazonia, pero las métricas e indicadores disponibles tienden a favorecer las estrategias hegemónicas y a invisibilizar soluciones que ya existen y que han evolucionado en la región ^3^. Una consecuencia ha sido la inadecuación de las políticas públicas tanto para satisfacer las necesidades de las poblaciones tradicionales como para satisfacer las nuevas necesidades que surgen en el espacio transformado.

Este Suplemento describe los resultados teóricos, metodológicos y analíticos del Proyecto Trayectorias, desarrollado desde una perspectiva de la Ciencia de Síntesis del Conocimiento. La propuesta de un Centro de Síntesis es promover un entorno interdisciplinario que favorezca la integración de teorías, métodos y datos en diferentes escalas espaciales o temporales, con el objetivo de contrarrestar la especialización científica. Cuando tiene éxito, la síntesis reúne temas, especialidades o disciplinas de maneras innovadoras, abriendo nuevas esferas de investigación e innovando para enfrentar los desafíos socioambientales ^4^. En el ambiente creativo y favorable proporcionado por el primer Centro de Síntesis de Biodiversidad y Servicios Ecosistémicos Brasileño (SinBiose, iniciativa del Consejo Nacional de Desarrollo Científico y Tecnológico, Tecnológico - CNPq, por sus siglas en Portugués), investigadores de varias instituciones de investigación brasileñas se reunieron para pensar en las siguientes preguntas rectoras: ¿Cómo se relacionan las diferentes trayectorias socioambientales con la salud humana y ambiental en el bioma amazónico? ¿En qué escenarios la salud humana y la salud ambiental son amplificadas simultáneamente por las mismas políticas públicas? ¿En qué escenarios se requieren políticas públicas para reducir los costos para el medio ambiente o para las comunidades locales derivados de los cambios en el uso de la tierra?

Las discusiones teóricas iniciales, resumidas en Codeço et al. ^5^, buscan ver el medio ambiente y la salud desde el concepto de trayectoria tecnoproductiva, que es la clasificación de la economía rural amazónica propuesta por el profesor economista Francisco Costa ^6^. Diferentes trayectoria tecnoproductiva definen relaciones específicas entre producción y naturaleza que emergen de lógicas, conocimientos y tecnologías de producción que incorporan o no un contexto ecológico en sus procesos. Estas relaciones con la naturaleza, a su vez, están asociadas con distintos paisajes epidemiológicos. En este Suplemento, el lector encontrará un testimonio del profesor Costa sobre los orígenes de la teoría de las trayectorias tecnoproductivas rurales en la Amazonia. Esta teoría ha servido de fundamento para la producción de una base de datos para los municipios de la Amazonia Legal, que relaciona las trayectoria tecnoproductiva con indicadores ambientales de uso y transformación del paisaje, indicadores epidemiológicos que miden enfermedades sensibles al clima e indicadores socioeconómicos adaptados a la realidad rural y realidad urbana de la Amazonia ^7^. Los artículos de este Suplemento profundizan en el análisis de estos indicadores, en diferentes frentes y perspectivas, aunando el conocimiento de investigadores con experiencia en salud, medio ambiente, economía y sociedad, para generar una narrativa más integrada y consistente para explicar/modificar los escenarios que afectan los ecosistemas y la salud en la Amazonia.
